# Mapping the tumor microenvironment in bladder cancer and exploring the prognostic genes by single-cell RNA sequencing

**DOI:** 10.3389/fonc.2022.1105026

**Published:** 2023-01-19

**Authors:** Zhibin Chen, Dongmao Chen, Zhenfeng Song, Yifan Lv, Defeng Qi

**Affiliations:** Department of Urology and Andrology, Minimally Invasive Surgery Center, Guangdong Provincial KeyLaboratory of Urology, The First Affiliated Hospital of Guangzhou Medical University, Guangzhou, Guangdong, China

**Keywords:** bladder cancer, tumor macroenvironment, MIBC (muscle-invasive bladder cancer), NMIBC (non-muscle-invasive bladder cancer), prognosis gene, single-cell RNA-sequencing

## Abstract

Despite substantial advances in the treatment using immune checkpoint inhibitors (ICIs), the clinical expected therapeutic effect on bladder cancer has not been achieved, in which the tumor microenvironment (TME) occupies a notable position. In this research, 10X single-cell RNA-sequencing technology was conducted to analyze seven primary bladder tumor tissues (three non-muscle-invasive bladder cancer (NMIBC) and four muscle-invasive bladder cancer (MIBC)) and seven corresponding normal tissues adjacent to cancer; eight various cell types were identified in the bladder cancer (BC) TME, and a complete TME atlas in bladder cancer was made. Moreover, bladder cancer epithelial cells were further subdivided into 14 subgroups, indicating a high intra-tumoral heterogeneity. Additionally, the differences between NMIBC and MIBC were compared based on differential gene expression heatmap, copy number variation (CNV) distribution heatmap, Gene Ontology (GO) enrichment analysis, and Kyoto Encyclopedia of Genes and Genomes (KEGG) enrichment analysis. Weighted gene co-expression network analysis (WGCNA), protein–protein interaction (PPI) network mutual analysis, and the Kaplan–Meier survival prognosis analysis were used to identify six key genes associated with the prognosis of bladder cancer: VEGFA, ANXA1, HSP90B1, PSMA7, PRDX6, and PPP1CB. The dynamic change of the expression distribution of six genes on the pseudo-time axis was further verified by cell pseudo-time analysis.

## 1 Introduction

Bladder cancer (BC) is regarded as the second most malignant tumor in the urogenital system in the world, and there are approximately 430,000 newly diagnosed cases of BC and more than 165,000 deaths due to BC each year worldwide, with an increasing trend in morbidity and mortality. According to the statistics of new cancer cases worldwide in 2018, the global age-standardized incidence (per 100,000 people) was approximately 9.6 for men and approximately 2.4 for women (1, 2). Clinically, BC is mainly divided into two subtypes: non-muscle-invasive bladder cancer (NMIBC) and muscle-invasive bladder cancer (MIBC). On the basis of epidemiological statistics, 25% of patients were diagnosed with MIBC or metastatic bladder cancer at initial diagnosis, though approximately 60% of bladder cancer are NMIBC, and its 5-year survival rate is up to 90% (3, 4). According to the 2022 Guidelines for the Management of Bladder Cancer, NMIBC is mainly treated with transurethral resection of bladder tumor (TURBT), followed by the standard-of-care immunotherapy with Bacillus Calmette–Guérin (BCG) or intravesical chemotherapy (5). As to MIBC, radical cystectomy combined with radiotherapy, chemotherapy, and immunotherapy is mainly used (6). In recent years, the tumor microenvironment (TME) and relevant immune checkpoint inhibitors (ICIs), which target programmed cell death protein 1 (PD-1) and its ligands (PD-L1) and the cytotoxic T-lymphocyte-associated antigen 4 (CTLA-4), have gradually become a focus of research in cancer treatment (7, 8). Nevertheless, only a small portion of patients receiving ICI treatment had a significant therapeutic effect (9, 10). Studies have shown that the treatment effect of ICI in cancer patients is related to the type and proportion of tumor-infiltrating lymphocytes (TILs) in the TME and their stress response to the tumors, in which cytotoxic CD4+/CD8+ T cells, NK cells, and macrophages participate in direct tumor cell killing and may occupy a unique position in immunotherapy (11–13). However, not all BC types with similar TIL structures in the TME have predictable clinical effects for immune targeted therapy, which highlights the complexity of the underlying tumor-immune interactions. Previously, five expression subtypes of bladder cancer have been identified, including light-papillary, light-invasive, luminal, basal–squamous, and neuronal, in which only the basal–squamous subtype has the richest immune expression characteristics, including T-cell markers and inflammatory genes, resulting in particular sensitivity to ICI (14–16). The complexity and heterogeneity of the TME can be better understood in the observation of tumor types that have not responded well to immunotherapy so far. Therefore, it is of great significance to further explore the composition of tumor-infiltrating lymphocytes in the TME of BC and the molecular characteristics of different tumor phenotypes of bladder cancer and to identify potential prognostic and predictive biomarkers for improving targeted therapy in the TME.

In this research, we performed a 10X single-cell analysis of the transcriptome of 105,123 individual cells from bladder cancer and paracancerous normal tissue samples and generated a complete TME atlas in bladder cancer tissues. We combined copy number variation (CNV) and a series of algorithms to re-cluster single cells with similar transcript information and further divide bladder cancer epithelial cells into several cell subtypes to explore intra- and inter-tumor heterogeneity. In addition, we used weighted gene co-expression network analysis (WGCNA) and protein–protein interaction (PPI) network analysis to compare the differences between the MIBC group and the NMIBC group and to identify prognostic genes associated with bladder cancer progression and transformation. A series of bioinformatics approaches were used to analyze their survival prognosis. These results promote the deeper exploration of high heterogeneity among patients and provide a theoretical basis for personalized therapy of BC.

## 2 Materials and methods

### 2.1 Patients and samples

All samples were attained from the First Affiliated Hospital of Guangzhou Medical University and the Cancer Hospital Affiliated with Guangzhou Medical University, Guangdong Province, China. Seven primary bladder tumor tissues (three NMIBC and four MIBC) along with seven corresponding normal tissues adjacent to cancer were involved in this research (**Table 1**). Informed consent for this research has been obtained from patients. All experimental processes have been approved by the School Evaluation Committee of Guangzhou Medical University.

**Table 1:** Clinical information of seven patients (the NC groups were the corresponding normal tissues adjacent to cancer).

Patients	Gender	Age	Histological grading	TNM stage	Lymph node metastasis	Recrudescence	Clinical stages
BC-01	Male	65	High-grade papillary urothelial carcinoma	T3xN0M0	No	No	MIBC
NC-01							
BC-04	Male	77	High-grade urothelial carcinoma	T4aN0M0	No	Recrudescence	
NC-04							
BC-05	Male	73	High-grade urothelial carcinoma with glandular differentiation	T4aN2Mx	Left and right pelvic lymph node metastasis	No	
NC-05							
BC-30	Male	57	Low-grade papillary urothelial carcinoma	T3aN0M0	No	No	
NC-30							
BC-11	Male	67	High-grade papillary urothelial carcinoma	TaN0M0	No	No	NMIBC
NC-11							
BC-24	Male	60	High-grade urothelial carcinoma	T1N0M0	No	No	
NC-24							
BC-27	Male	57	High-grade papillary urothelial carcinoma	T1N0M0	No	No	
NC-27							

MIBC, muscle-invasive bladder cancer; NMIBC, non-muscle-invasive bladder cancer.

### 2.2 Single-cell suspension preparation

The bladder cancer tissues and adjacent normal tissues were taken for immediate histological treatment. Each sample is cut into small pieces with a diameter of <2 mm, ground, sieved with 300 mesh nylon mesh, and washed continuously with phosphate-buffered saline (PBS) to obtain a suspension. The samples were then placed in a shaker at 37°C; appropriate trypsin, collagenase II, and DNA enzyme were added; they were incubated for 1 h. Then, PBS was added to dilute the suspension, and the suspension was filtered using a 40-μm cell sieve. After centrifugation at 1,500 rpm for 5 min, the supernatant was removed, and the cells were washed twice with PBS. Then, 1 ml of red blood cell lysate was added for 5 min at room temperature. Next, 10 ml of PBS was added to the test tube and centrifuged at 1,500 rpm for 10 min. After the last centrifugation, the supernatant was poured out, and a complete RPMI 1640 culture medium was added to resuspend the cells and mixed well (17).

### 2.3 Droplet-based single-cell sequencing

A small amount of single-cell suspension was taken, 0.4% trypan blue dye of equal volume was added, Countless^®^ II Automated Cell Counter was used to count the cells, and the concentration of living cells was adjusted to the ideal concentration (1,000–2,000 cells/μl). The gel beads containing barcode information were combined with the mixture of cells and enzymes, entered the reservoir, and were separated by oil to form GEMs (Gel Beads in Emulations). After that, the gel beads were dissolved, the captured sequence containing the barcode sequence was released, the cDNA fragment was reverse-transcribed, and the sample was labeled. The gel beads and the oil drops were broken, and the cDNA was used as the template for PCR amplification. The products of all GEMs were mixed to construct a standard sequencing library. The cDNA was cut into several fragments of approximately 200–300 bp by cDNA enzyme, and then the DNA library was obtained by PCR amplification after conventional second-generation sequencing library construction steps such as end repair, adding tail A, sequencing connectors P5 and P7, and sample index. Finally, the high flux sequencing of constructed DNA library was conducted by the Illumina sequencing platform.

### 2.4 Quality control and single-cell subgroup classification

The software Cell Ranger was used to filter, compare, quantify, and identify the recovered cells from the original data and finally obtained the gene expression matrix of each cell. Subsequently, Seurat was used for further abnormal cell filtration, in which the cell filtration conditions followed that the number of genes; unique molecular identifiers (UMIs) in a single cell should be at 300–800 and 600–40,000. Moreover, the proportion of mitochondrial gene expression is less than 25%. After low-quality cells were removed, the LogNormalize method of the Normalization function was used to homogenize the data expression through the Seurat software, and high characteristic genes between cells were found using the vst method of the FindVariableFeatures function. Then, canonical correlation analysis (CCA) was performed on all samples to search for the mutual nearest neighbor (MNN) between cells in order to build a mapping relationship between cells, which can facilitate cell clustering and exclude standardization of transcript differences caused by batch effects. Furthermore, the ScaleData function of the Seurat software was performed to normalize the Z-score of the data so that the principal component analysis (PCA) can be conducted with the use of the homogenized expression value. Finally, the clustering algorithm based on graph theory in Seurat software was used to cluster and group the cells.

### 2.5 Assessment of CNV in cancer cells

The CNV analysis was used to detect CNV in epithelial cells of bladder cancer tissues. Fibroblast colonies were used as the control group, default parameters were used to identify cancer cells and normal cells, and CNV heterogeneity between different samples was compared.

### 2.6 Differentially expressed gene analysis and pathway enrichment

Seurat’s rank sum test was used to analyze the gene differential expression of different cell groups. |FC| > 2 and adjusted p-value <0.05 were considered as the cutoff criteria. Gene Ontology (GO) and Kyoto Encyclopedia of Genes and Genomes (KEGG) pathway enrichment analyses were performed on the above differentially expressed genes (DEGs).

### 2.7 Weighted gene co-expression network analysis

WGCNA was constructed for epithelial cell subsets of the MIBC group and NMIBC group using the R language package to construct a hierarchical clustering tree in order to select the gene modules related to BC progression.

### 2.8 Protein–protein interaction network construction

Through the STRING database, the information on the protein interaction relationship of upregulated genes can be obtained so as to construct a network map of protein interaction for the screened gene set that is significantly related to the development of bladder cancer.

### 2.9 Pseudo-time analysis

In order to locate the differentiation status of bladder cancer at different stages, a pseudo-time analysis was performed with Monocle3 to determine the trajectory relationship between cell type and bladder cancer stage. Through further detection of the time axis differential gene function module, the dynamic changes in the expression distribution of differential genes on the quasi-time axis are displayed.

## 3 Results

### 3.1 Single-cell clustering and cell type identification in bladder cancer

**Figure 1:**
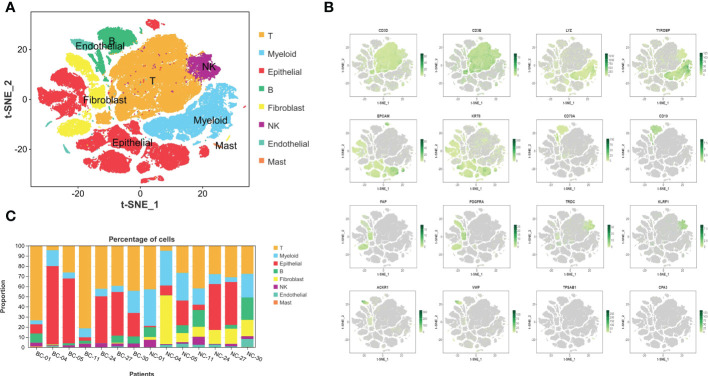
The complex composition of the tumor microenvironment in bladder cancer and corresponding normal tissues adjacent to cancer. **(A)** Eight kinds of basic infiltrated cell types in TME. **(B)** The expression and distribution of identification markers: T cells: CD3D and CD3E, myeloid cells: LYZ and TYROBP, epithelial cells: EPCAM and KRT8, B cells: CD79A and CD19, fibroblast cells: FAP and PDGFRA, NK cells: TRDC and KLRF1, endothelial cells: ACKR1 and VWF, and mast cells: TPSAB1 and CPA3. **(C)** Varying constituent ratio of infiltrated cell types in different BC and corresponding normal tissues. TME, tumor microenvironment; BC, bladder cancer.

After quality control and elimination of batch effect between different samples (18), 131,993 cells were captured. Then, 105,123 high-quality cells were obtained after filtration by Seurat and double finder, and 105,123 single cells were clustered into eight main clusters (**Figure 1A**). Cell types were identified according to Singler and screened marker genes with significant differences: T cells (CD3D and CD3E), myeloid cells (LYZ and TYROBP), epithelial cells (EPCAM and KRT8), B cells (CD79A and CD19), fibroblast cells (FAP and PDGFRA), NK cells (TRDC and KLRF1), endothelial cells (ACKR1 and VWF), and mast cells (TPSAB1 and CPA3) (**Figure 1B**). **Figure 1C** shows that the composition of TIL reveals obvious heterogeneity in different bladder cancer tumors. In bladder cancer tissues, T cells and B cells show a phenomenon of expansion, while myeloid cells and fibroblasts are reduced compared with normal tissues.

### 3.2 Intra-tumoral and inter-tumoral heterogeneity in bladder cancer

**Figure 2:**
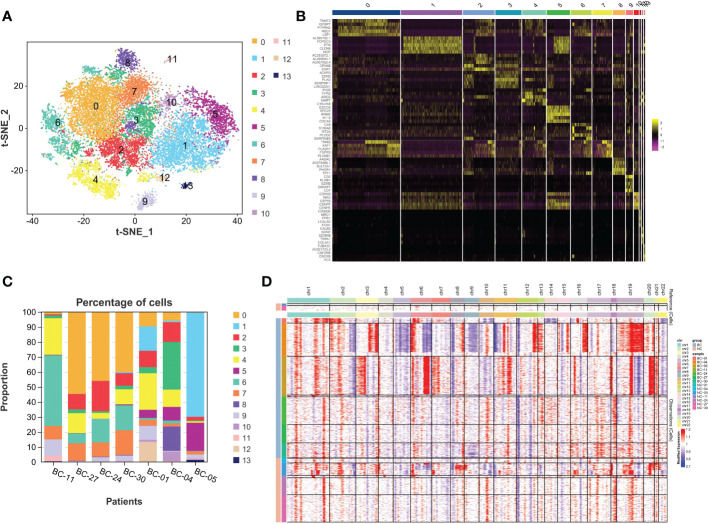
The intra-tumor and inter-tumor heterogeneity of BC. **(A)** The malignant epithelial cells were extracted and re-clustered into 14 clusters. **(B)** Top 5 marker genes of 14 malignant cell clusters are displayed in the form of heatmaps. **(C)** Varying proportions of 14 cell types between BC groups. **(D)** The CNV distribution heatmap in bladder cancer and corresponding normal tissues. BC, bladder cancer; CNV, copy number variation.

To further explore tumor heterogeneity, epithelial cells in the bladder cancer group were divided into 14 subtypes of malignant cells (**Figure 2A**). Interestingly, widespread and significant heterogeneity was found in tumor cells, even within the same tumor type. When characterizing different subtypes, the top 5 marker genes are represented in the heatmap (**Figure 2B**). In order to more distinctly demonstrate intra- and inter-tumoral heterogeneity, the composition ratio of various epithelial cell subsets in each BC patient was described (**Figure 2C**). It is obvious that in BC-05 patients, cluster 1 accounts for more than 70%, constituting the primary cancer population, while it was discovered to be small in other BC patients, especially in the NMIBC group. According to the clinical information of the patients, it was found that the BC-05 patients had a relapsing type, and the clinical stage was T4aN2M0. In addition, BC-11 patients were mainly composed of cluster 6. In addition, with the progress of the clinical stage, the proportion of cluster 0 decreased gradually. Thus, the proportion of cell subsets varied between cases, indicating intra- and inter-tumor heterogeneity. CNV is known to be widespread in tumor cells. Consequently, the heterogeneity of CNV in different BC patients was further studied. As shown in **Figure 2D**, the MIBC group as a whole showed more significant CNV than the NMIBC group. In addition, CNV differences among different individuals in the same group were also sharp; for example, BC-04 patients showed a high CNV increase on chromosome 19 with significant copy number loss on chromosome 5, while BC-05 patients showed CNV amplification on chromosomes 6 and 20. Therefore, the heterogeneity of BC is represented not only in the composition of various cell types but also in the modality of transgenation.

### 3.3 Differences between MIBC and NMIBC

**Figure 3:**
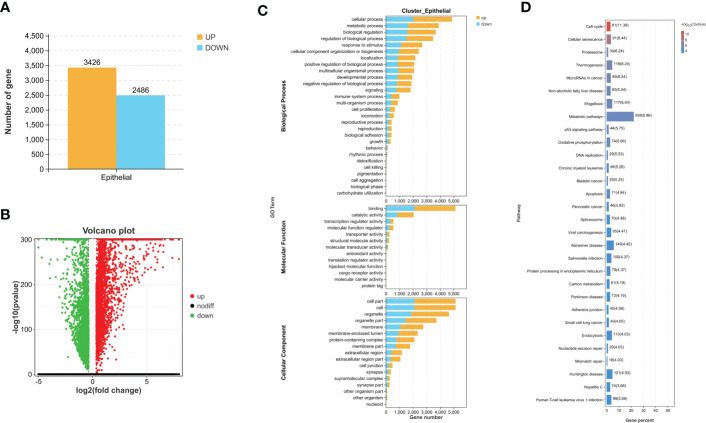
Difference comparison between MIBC and NMIBC. **(A)** The number of upregulated DEGs and downregulated DEGs between MIBC and NMIBC. **(B)** The volcano plot of DEGs. **(C)** Enriched GO functions of DEGs in malignant epithelial cells. **(D)** Highly enriched KEGG pathway of DEGs in malignant epithelial cells. MIBC, muscle-invasive bladder cancer; NMIBC, non-muscle-invasive bladder cancer; DEGs, differentially expressed genes; GO, Gene Ontology; KEGG, Kyoto Encyclopedia of Genes and Genomes.

**Figure 4:**
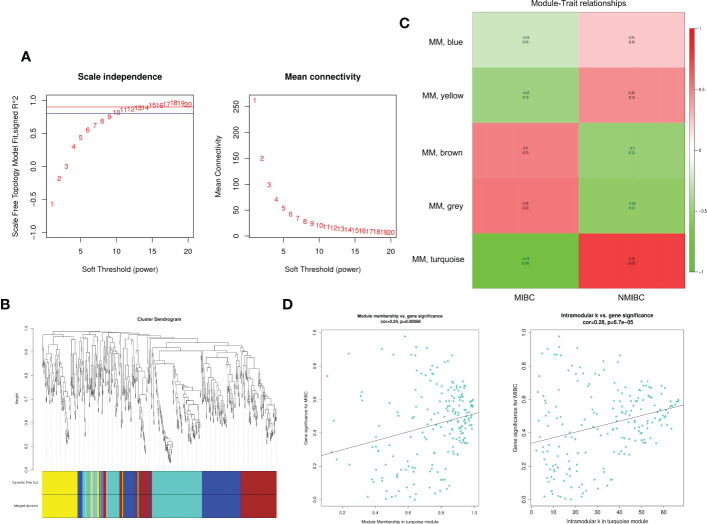
The exploration for module relevant to invasion depth of bladder cancer by WGCNA. **(A)** The appropriate power value was selected according to the scale-free network principle. We consider the minimum power value (p = 10) as the parameter for subsequent analysis when the correlation coefficient (scale-free fit index R^2^ (R^2^ = 0.80)) reaches the platform stage. **(B)** Dynamic Tree Cut is a dendrogram of the DEGs divided according to the clustering results. **(C)** The heatmap reveals the correlation between different gene modules and clinical features (MIBC and NMIBC). **(D)** Pearson’s correlation analysis between GS, MM, and K.n values of turquoise module and MIBC. WGCNA, weighted gene co-expression network analysis; DEGs, differentially expressed genes; MIBC, muscle-invasive bladder cancer; NMIBC, non-muscle-invasive bladder cancer.

**Figure 5:**
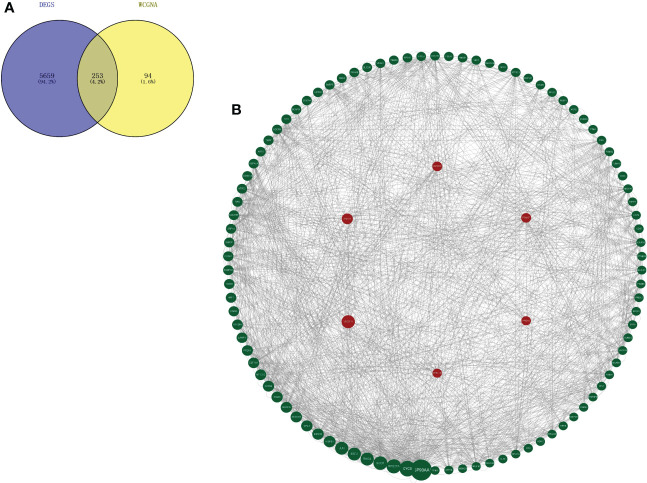
Key marker genes associated with the prognosis were explored. **(A)** Genes that overlap in turquoise module from WGCNA and DEGs between MIBC and NMIBC. **(B)** PPI diagram of top 100 genes based on the betweenness values by Cytoscape and the six hub genes is remarked with a red circle as background. WGCNA, weighted gene co-expression network analysis; DEGs, differentially expressed genes; MIBC, muscle-invasive bladder cancer; NMIBC, non-muscle-invasive bladder cancer; PPI, protein–protein interaction.

Through differential expression analysis, 5,912 genes were identified as DEGs in the aggregate (**Figure 3A**), which consist of 3,426 significantly upregulated genes and 2,486 markedly downregulated genes (**Figure 3B**). In the biological process, DEGs are mainly abundant in the cells, metadata, regulation of the biological process, and response to stimulus, which primarily involve the molecular function of cell binding and catalytic activity (**Figure 3C**). In the KEGG pathway, it is shown that DEGs were mainly enriched in the metabolic pathway, cell cycle, cellular senescence, thermogenesis, P53 signaling pathway, oxidative phosphorylation, microRNAs relevant to cancer, etc. (**Figure 3D**). Next, WGCNA was conducted to determine the gene modules closely related to the invasion depth of BC. During the construction of the co-expression network, the minimum power value of 10 was considered as a parameter for subsequent analysis when the correlation coefficient reaches the plateau period (or greater than 0.8) (**Figure 4A**). Subsequently, five different modules were identified (**Figure 4B**), in which the turquoise module was significantly bound up with the invasion depth of bladder cancer (r = 0.75, p = 0.05), on the basis of the correlation coefficient and p-value (**Figure 4C**). Pearson’s correlation analysis was further conducted on the GS, MM, and K.n values of the turquoise module and bladder cancer. The gene within the turquoise module was found to be highly correlated with the stage of bladder cancer (MM-GS, COR = 0.24, p = 0.00068; K.N-GS, COR = 0.28, p = 6.7e−0.5) (**Figure 4D**). Finally, 253 common genes of both DEG and WGCNA turquoise module were selected (**Figure 5A**), and the expression matrix was constructed for further analysis.

### 3.4 Identification of hub genes

A total of 253 genes were selected for PPI analysis. The top 100 genes were picked out based on the betweenness centrality (BC) value by Cytoscape software, and the PPI network diagram was drawn (**Figure 5B**). The Kaplan–Meier survival prognosis analysis was performed on the top 100 genes, and 6 genes related to the survival prognosis of bladder cancer were finally screened out: VEGFA, ANXA1, HSP90B1, PSMA7, PRDX6, and PPP1CB.

### 3.5 The six genes related to the prognosis of bladder cancer: VEGFA, ANXA1, HSP90B1, PSMA7, PRDX6, and PPP1CB

**Figure 6:**
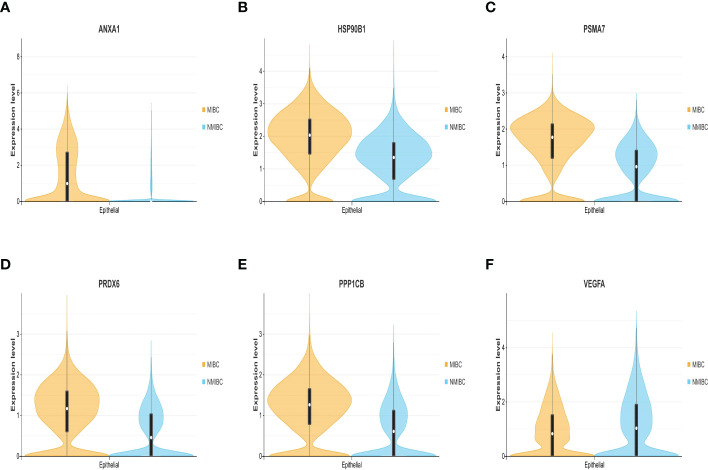
**(A–F)** The expression level and density of ANXA1, HSP90B1, PSMA7, PRDX6, PPP1CB, and VEGFA genes between MIBC and NMIBC in epithelial cluster. MIBC, muscle-invasive bladder cancer; NMIBC, non-muscle-invasive bladder cancer.

**Figure 7:**
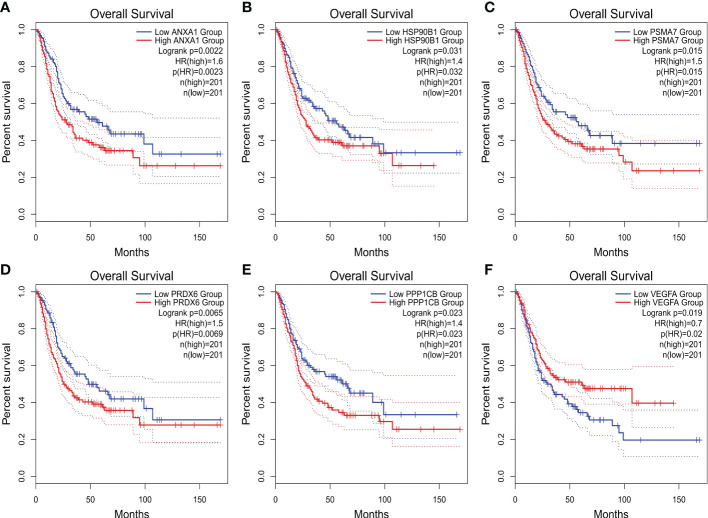
**(A–F)** The overall survival (OS) curve of ANXA1, HSP90B1, PSMA7, PRDX6, PPP1CB, and VEGFA genes.

**Figure 8:**
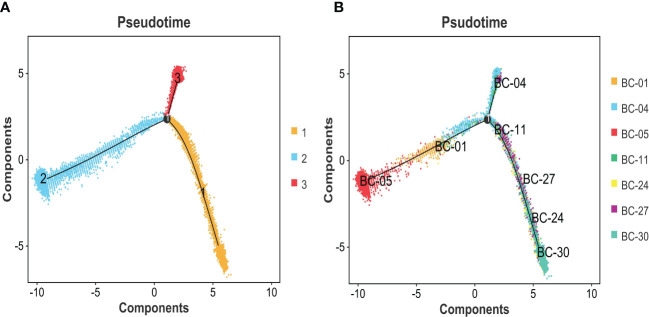
**(A)** Pseudotime analysis was used to construct the trajectory of epithelial cell differentiation in BC groups, divided into three separate evolutionary tracks. **(B)** Combined with the clinical grading of patients, it is obvious that two trajectory branches are consistent with MIBC, while one trajectory branch corresponds to NMIBC. BC, bladder cancer; MIBC, muscle-invasive bladder cancer; NMIBC, non-muscle-invasive bladder cancer.

**Figure 9:**
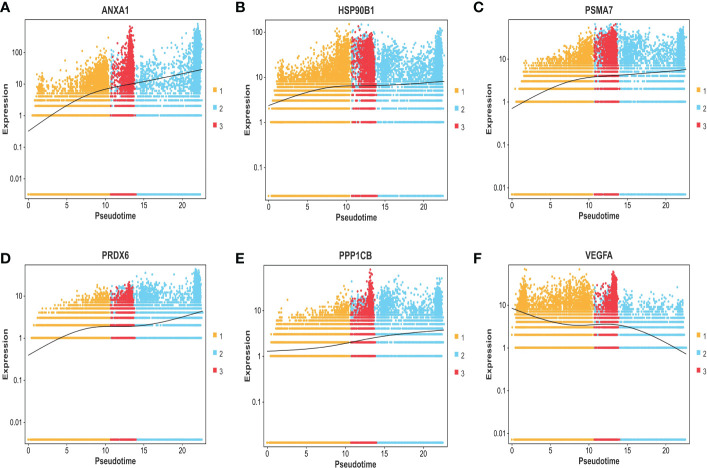
**(A–F)** The expression trend of ANXA1, HSP90B1, PSMA7, PRDX6, PPP1CB, and VEGFA genes based on the pseudo-time axis.

The expression differences of the six genes were further compared between MIBC and NMIBC. As shown in **Figures 6A–E**, the expression levels of ANXA1, HSP90B1, PSMA7, PRDX6, and PPP1CB are all higher in the MIBC groups (ANXA1, p < 0.01; HSP90B1, p < 0.001; PSMA7, p < 0.001; PRDX6, p < 0.001; PPP1CB, p < 0.001), while it was the opposite in VEGFA gene, high expression in the NMIBC group (p = 4.90250067170868e−213) (**Figure 6F**). To evaluate the correlation between these genes and patient prognosis, the Kaplan–Meier survival prognosis analysis was performed based on the GEPIA2 platform. The results showed that the lower expression levels of ANXA1, HSP90B1, PSMA7, PRDX6, and PPP1CB are associated with better prognosis (ANXA1, p = 0.0022, HR = 1.6, p(HR) = 0.0023; HSP90B1, p = 0.031, HR = 1.4, p(HR) = 0.032; PSMA7, p = 0.015, HR = 1.5, p(HR) = 0.015; PRDX6, p = 0.0065, HR = 1.5, p(HR) = 0.0069; PPP1CB, p = 0.023, HR = 1.4, p(HR) = 0.023) (**Figures 7A–E**), while higher expression of VEGFA gene was linked to better prognosis of bladder cancer (VEGFA, p = 0.019, HR = 0.7, p(HR) = 0.02) (**Figure 7F**). Meanwhile, in order to further track the dynamic changes of these six genes in the progression of bladder cancer, a pseudo-time series analysis was used. Combined with the clinical staging of patients, it can be found that the clinical progress of bladder cancer presents a certain time correlation. Meanwhile, relapsing bladder cancer shows a new differentiation trajectory compared with common MIBC (**Figures 8A, B**). The expression of ANXA1, HSP90B1, PSMA7, PRDX6, and PPP1CB genes increased with the progression of clinical stages (**Figures 9A–E**), while the expression of VEGFA gene decreased gradually (**Figure 9F**).

In summary, ANXA1, HSP90B1, PSMA7, PRDX6, and PPP1CB genes with high expression are bound up with a poor prognosis of BC. ANXA1, HSP90B1, PSMA7, PRDX6, and PPP1CB genes with high expression may promote the progression of NMIBC to MIBC. The high expression of VEGFA gene may inhibit the progression of NMIBC to MIBC.

## 4 Discussion

BC is one of the most widespread genitourinary malignancies, which had resulted in more than 165,000 deaths globally. Although TURBT amalgamated with intravesical chemotherapy, even with standard treatment, could achieve certain curative effects in NMIBC, 30% of patients recur after TURBT with the risk of progression to MIBC or even metastatic bladder cancer (19). In recent years, the rapid advancement of single-cell sequencing technology has provided a new vision for cancer research, in which the TME is believed to occupy a considerable position in the occurrence and development of BC. The TME is a complicated system composed of tumor cells, immune, inflammatory cells and fibroblasts relevant to tumors, neighboring interstitial tissues, microvessels, and various cytokines and chemokines, in which cancer cells can shape their microenvironment through intercellular interactions (20).

The formation and evolution of the TME are closely linked to the biological behavior of the tumor and the prognosis of the disease. However, there are still few studies on this technique in bladder cancer. Therefore, considering the vital role of the TME in cancer progression and development, it is necessary for us to explore the composition of the TME of bladder cancer and explore potential prognostic biomarkers to provide clues for immunotherapy and targeted therapy for bladder cancer.

First, high-throughput transcriptome sequencing analysis was conducted on 105,123 single cells from three NMIBC, four MIBC, and corresponding seven paracancerous normal tissues. Based on a series of algorithms, single cells with similar transcriptional information were clustered, and 33 cell types were identified by consensus, which demonstrates the diversity, complexity, and heterogeneity of cell types in the tumor microenvironment. Then, the basic cell types of the bladder cancer microenvironment were identified by combining differential genes between subtypes and markers confirmed in related literature. The bladder cancer microenvironment is mostly composed of T cells, myeloid cells, epithelial cells, B cells, fibroblast cells, NK cells, endothelial cells, and mast cells, which leads to a complete TME atlas of bladder cancer.

Furthermore, according to the differences in marker genes, bladder cancer epithelial cells from the MIBC group were reclustered into 14 cell subtypes, and the expressions of marker genes in different subtypes were shown by a differential gene expressions heatmap. It was found that in patients with BC-05 (T4aN2M0), cluster 1 accounted for more than 70%, which constitutes the dominant tumor population, suggesting that the cells in cluster 1 are associated with lymph node metastasis of bladder cancer. Then, CNV variation was compared between different bladder cancers and between MIBC and NMIBC, showing that intra-tumor heterogeneity is reflected in not only composition but also the form of gene mutations.

GO and KEGG analyses showed that the differential genes between MIBC and NMIBC were mainly related to immune response, P53 signaling pathway, and oxidative phosphorylation. Subsequently, through WGCNA analysis, turquoise modules that were significantly associated with the occurrence and development of bladder cancer were identified. Finally, 253 common genes that were both DEGs and WGCNA modules were selected, and PPI analysis was conducted on 253 genes. According to BC values and the Kaplan–Meier survival curve, six genes related to the occurrence and development of bladder cancer were screened out: VEGFA, ANXA1, HSP90B1, PSMA7, PRDX6, and PPP1CB. It has been reported that VEGFA is mainly secreted by tumor cells, endothelial cells, and infiltrating myeloid cells in the tumor microenvironment, indicating hypoxia in the local microenvironment. VEGFA can induce angiogenesis by activating the VEGFR2-mediated angiogenesis response (21). Studies have shown that VEGFA can affect the infiltration of CD163+ TAM in bladder cancer (22), and the changes in VEGF receptor expression are linked to the disease staging and recurrence of bladder cancer (23). Pignot et al. found that T3–T4 bladder cancer with VEGFA overexpression is most likely to benefit from anti-angiogenic therapy (24). Therefore, VEGFA is a good prognostic factor and a promising therapeutic target for MIBC.

According to related studies, Annex A1 (ANXA1) is a calcium-ion and phosphatide-binding protein that not only regulates inflammation and immunity (for example, ANXA1 can enhance the inhibitory effect of Treg cells in the TME) (25) but also regulates cell proliferation and apoptosis. Previous studies have shown that high ANXA1 expression is bound up with a bad prognosis in patients with colorectal cancer and breast cancer (26). In addition, ANXA1 is significantly overexpressed in bladder cancer than corresponding normal tissues and may be related to the infiltration of immune cells in the TME (27). In the present research, the expression level of ANXA1 was significantly higher in MIBC than in NMIBC, with statistical significance. Meanwhile, high ANXA1 expression predicted the progression and poor prognosis of bladder cancer. Heat shock protein 90kDaβ1 (HSP90B1), belonging to the heat shock protein (HSP) 90 family, is a chaperone for a variety of stress-inducing molecules, such as Toll-like receptors (TLRs) and LRP6 (28). Previous studies have shown that HSP90B1 maintains a delicate balance between cancer cell survival and death by regulating endoplasmic reticulum (ER)-related pro-apoptotic mechanisms. Relevant studies have shown that high expression of HSP90B1 usually predicts poor overall survival and disease-free survival of tumor patients, such as adrenal cortical carcinoma, renal papillary cell carcinoma, lung adenocarcinoma, and non-small cell lung cancer (NSCLC) (29, 30). In this study, the pseudo-time series analysis confirmed that the expression level of HSP90B1 was gradually increased during the progression of bladder cancer, indicating a poor prognosis. Proteasome subunit α7 type (PSMA7), an α-type subunit of the 20S proteasome core complex, is involved in protein degradation through the ubiquitin–proteasome pathway (UPP) and plays a vital role in the regulation of cell differentiation and apoptosis (31). Recent studies have shown that PSMA7 silencing can promote apoptosis by inhibiting UPP signaling pathway, as well as inhibiting cervical cancer cell proliferation and VEGF expression (32). This study also reveals that PSMA7 is a potential therapeutic target for bladder cancer, with overexpression in MIBC and poor prognosis. Peroxide-reducing protein 6 (Prdx6) belongs to the peroxidase family, which not only is an important enzymatic antioxidant but also has the activity of phospholipase A2. Prdx6 participates in the signal transduction between cells, for which it can stifle cell apoptosis and activate cell proliferation (33). The expression of PRDX6 can promote the proliferation, migration, and invasion of colorectal cancer cells (34). To date, PRDX6 is relatively insufficient in the study of bladder cancer. Our research indicates that PRDX6 can promote the progress of bladder cancer and is associated with poor prognosis, while the specific mechanism of action needs further study. Protein phosphatase-1 catalyzed subunit β (PPP1CB) is related to the generation and differentiation of adipocytes, which is relatively scarce in cancer studies. However, this study also found that the high expression of PPP1CB is relevant to the poor prognosis of BC, which revealed that the genes related to fat metabolism are another focus of bladder cancer treatment.

In conclusion, this study reveals the infiltrated cell composition in the tumor microenvironment of bladder cancer and expounds the high heterogeneity intra- and inter-tumor and the correlation between genes VEGFA, ANXA1, HSP90B1, PSMA7, PRDX6, and PPP1CB; the invasion degree of BC was elaborated, as well as the dynamic changes in the progression of BC, which confirms that they were highly correlated with the prognosis of the BC. Therefore, genes VEGFA, ANXA1, HSP90B1, PSMA7, PRDX6, and PPP1CB may be new targets for anti-tumor effects in the future.

## 5 Study strengths and limitations

The primary aim of this study was to produce a complete TME atlas in bladder cancer tissues, clarify the composition of tumor-infiltrating cells types in the tumor microenvironment, elaborate the high heterogeneity in bladder cancer, and further pick out the key genes related to the prognosis of bladder cancer. The main limitation of this study is the lack of basic experimental verification. Therefore, further experiments are necessary to verify the role of prognostic genes in bladder cancer and the potential mechanism of action.

## Data availability statement

The raw sequence data of Single-cell RNA-sequencing presented in the study are deposited in the Genome Sequence Archive (Genomics, Proteomics & Bioinformatics 2021) in National Genomics Data Center (Nucleic Acids Res 2022), China National Center for Bioinformation/Beijing Institute of Genomics, Chinese Academy of Sciences (accession number: HRA003620) that are publicly accessible at https://ngdc.cncb.ac.cn/gsa-human. The other relevant data are available in the article, supplementary information, or from Protein-Protein Interaction Networks STRING (https://cn.string-db.org/), Gene Expression Profiling Interactive Analysis GEPIA2 (http://gepia2.cancer-pku.cn/#index)/**Supplementary Material**.

## Ethics statement

The studies involving human participants were reviewed and approved by Medical Ethics Committee of the First Affiliated Hospital of Guangzhou Medical University. The patients/participants provided their written informed consent to participate in this study. Written informed consent was obtained from the individual(s) for the publication of any potentially identifiable images or data included in this article.

## Author contributions

ZC: sample collection, project development, data analysis, and manuscript writing. DC: data collection and analysis. All authors contributed to the article and approved the submitted version.

## Supplementary material

The Supplementary Material for this article can be foundonline at: https://www.frontiersin.org/articles/10.3389/fonc.2022.1105026/full#supplementary-material
